# The effect of exercise and disease status on mobilization of anti-tumorigenic and pro-tumorigenic immune cells in women with breast cancer

**DOI:** 10.3389/fimmu.2024.1394420

**Published:** 2024-06-24

**Authors:** Tiia Koivula, Salla Lempiäinen, Joona Neuvonen, Jooa Norha, Maija Hollmén, Carl Johan Sundberg, Helene Rundqvist, Heikki Minn, Petteri Rinne, Ilkka Heinonen

**Affiliations:** ^1^ Turku PET Centre, University of Turku and Turku University Hospital, Turku, Finland; ^2^ MediCity Research Laboratory, University of Turku, Turku, Finland; ^3^ Department of Physiology and Pharmacology, Karolinska Institutet, Stockholm, Sweden; ^4^ Department of Learning, Informatics, Management and Ethics, Karolinska Institutet, Stockholm, Sweden; ^5^ Department of Laboratory Medicine, Karolinska Institutet, Stockholm, Sweden; ^6^ Department of Oncology and Radiotherapy, Turku University Hospital, Turku, Finland; ^7^ Institute of Biomedicine, University of Turku, Turku, Finland

**Keywords:** acute exercise, physical activity, breast cancer, white blood cell, immune cell, immunity

## Abstract

**Background:**

Mobilization of certain immune cells may improve the ability of the immune system to combat tumor cells, but the effect of acute exercise on mobilizing immune cells has been sparsely investigated in cancer patients. Therefore, we examined how acute exercise influences circulating immune cells in breast cancer patients.

**Methods:**

Nineteen newly diagnosed breast cancer patients aged 36–68 performed 30 minutes of moderate-intensity exercise with a cycle ergometer. Blood samples were collected at various time points: at rest, at 15 (E15) and 30 minutes (E30) after onset of the exercise, and at 30 and 60 minutes post-exercise. We analyzed several immune cell subsets using flow cytometry.

**Results:**

Acute exercise increased the number of total leukocytes, neutrophils, lymphocytes, monocytes, basophils, total T-cells, CD4^+^ T-cells, T helper (Th) 2-cells, Th 17-cells, CD8^+^ T-cells, CD4^-^CD8^-^ T-cells, CD56^+^ natural killer (NK) cells, and CD14^-^CD16^+^ monocytes. Many of the changes were transient. Proportions of NK-cells and CD8^+^ T-cells increased, while the proportion of myeloid derived suppressor cells (MDSCs) reduced, and proportion of regulatory T-cells remained unchanged by exercise. Several associations were detected between cell mobilizations and disease state. For instance, tumor size correlated negatively with NK cell mobilization at E15, and progesterone receptor positivity correlated negatively with CD8^+^ T-cell mobilization.

**Conclusion:**

The findings show that the proportions of CD8^+^ T-cells and NK cells increased and the proportion of MDSCs proportion decreased in breast cancer patients after 30-minute exercise, suggesting a change in the profile of circulating immune cells towards more cytotoxic/anti-tumorigenic. The mobilization of some immune cells also appears to be related to the disease state.

## Introduction

Physical exercise has been found to prevent cancer, reduce the side effects of cancer treatments and improve prognosis and patients’ quality of life. For example, the likelihood of being diagnosed with breast cancer is 13% lower in people who are most physically active in their leisure time versus the ones who are least (physically) active (1). Moreover, cancer-specific mortality is 31% lower in physically active people post-diagnosis compared to sedentary ones (1). Traditionally, patients were advised to rest during cancer treatments, but due the positive effect of exercise on cancer and treatment related side effects as well as associations with a better prognosis, the view has turned. For example, exercise is the only effective way to reduce treatment related fatigue. Today, exercise is recommended for cancer patients similar to individuals without cancer (2). The current understanding is that many of the health benefits of exercise are attributed to the reduction of cancer risk factors such as obesity, hormonal levels and poor immune surveillance (3).

Tumor immune surveillance is a balance between immune cells that promote tumor progression (pro-tumorigenic cells) and cells that promote tumor rejection (anti-tumorigenic cells) (4). Studies in animal cancer models have shown that exercise shrinks tumors by enhancing the tumor infiltration of anti-tumorigenic cells, thus natural killer (NK) and cytotoxic T cells (5, 6). Furthermore, exercise reduces the accumulation and recruitment of pro-tumorigenic myeloid derived suppressor cells (MDSC) and regulatory T cells to tumors in mice with breast cancer (7, 8). In breast cancer patients, it has been demonstrated that higher CD8^+^ T cell count in tumor microenvironment (TME) is associated with a better prognosis and longer overall survival (9, 10). The survival rate of cancer patients has also been linked to the presence of NK cells at TME (11). On the contrary, the high number of infiltrating regulatory T cells and MDSCs is associated with more aggressive breast cancer subtypes and with worse overall survival (12–14).

Acute physical exercise is a powerful stimulus for mobilizing immune cells in healthy individuals, but the phenomenon is less investigated in cancer patients. In healthy individuals, the number of circulating neutrophils, lymphocytes, and monocytes increase in response to different types of exercise (15–18). Previously, we showed that acute 10-minute light-to-moderate-intensity exercise increases the number of total leukocytes, CD8^+^ T cells, CD19^+^ B cells, CD16^+^ NK cells, and CD14^+^CD16^+^ monocytes in the circulation of newly diagnosed breast cancer patients (13) and fairly similar responses were seen in lymphoma patients (19). These studies, however, did not include pro-tumorigenic cells, for example MDSCs or regulatory T cells. Thus, the aim of this study was to investigate immune cell responses to a 30-minute acute exercise session with moderate intensity in breast cancer patients. Specifically, we used flow cytometry to assess responses in circulating leukocytes, neutrophils, lymphocytes (B cells, T cells, NK cells), monocytes, eosinophils, basophils, and MDSCs at multiple time points during and after acute exercise. We wanted to investigate both the number of these cells, but also their proportions, and a profile between anti-tumorigenic and pro-tumorigenic cells. In addition, we aimed to examine whether oncologic disease status affects immune cell mobilization by exercise.

## Materials and methods

This study was conducted at the Turku PET Centre, Turku, Finland between August 2021, and May 2022. The study was approved by the Ethics Committee of the Hospital District of Southwestern Finland (100/1801/2020). Good clinical practice and the Declaration of Helsinki were followed. The study was a part of a larger study and is registered in the international register of clinical trials (Clinicaltrials.gov NCT04990856).

### Participants

Nineteen women with newly diagnosed breast cancer were recruited in this study before the onset of cancer treatments. Participants were recruited from Turku University Hospital. Exclusion criteria were abnormal fatigue, anemia, or physical disability that would hinder the study procedures. All participants provided written informed consent.

### Exercise test

Each participant visited the study laboratory once after study enrollment. Strong physical exertion and alcohol and caffeine consumption were prohibited for at least 24 hours prior the study day. Participants conducted a 30-minute exercise with a cycle ergometer (Gymstick Vapor 10.0, Gymstick International Oy, Lahti, Finland) with cadence and resistance that they would be able to sustain for 30 minutes. Participants were allowed to change the resistance as many times as they liked during the exercise. However, 70% of age predicted maximal heart rate was calculated using the formula (220 – age) * 0.7 and participants were asked to reach that heart rate at some point during the exercise. Lactate concentration (Lactate Scout 4, EKF Diagnostics, Barleben, Germany), blood pressure (Apteq AE701f, Rossmax Swiss GmbH, Berneck, Switzerland), and heart rate (Palmsat 2500, Nonin, Plymouth, USA) were measured at the same time points as the blood samples were drawn, and heart rate was also measured continuously during the exercise with Polar H10 heart rate sensor (Polar Electro Oy, Kempele, Finland). Participants’ rating of perceived exertion (RPE) was determined with a Borg scale (6–20). Rate pressure product (RPP) was measured by multiplying systolic blood pressure by heart rate. Mean arterial pressure (MAP) was calculated as (diastolic blood pressure * 2 + systolic blood pressure)/3. Predicted energy expenditure (EE) during exercise was calculated with the following formula: 0.744 + 0.0216 * (HRR) + 0.00699 * (weight) + 0.00102 * (HRR) * (weight) (20), HRR being heart rate reserve. Participants were allowed to drink water *ad libitum* during and after the exercise.

### Blood sampling and hematology analysis

Prior to starting the exercise bout, an intravenous catheter was placed in the antecubital vein for repeated blood sampling. The first blood sample was drawn after a short period of supine rest before the exercise. The second blood sample was drawn during the exercise, at 15-minute timepoint (E15) and the third at 30-minute timepoint when they were finishing the exercise (E30). Fourth and fifth blood samples were drawn during supine recovery, at 30-minutes post-exercise (P30) and at 60-minutes post-exercise (P60). All blood samples were collected in EDTA-tubes (BD Biosciences, San Jose, USA). We were able to collect all blood samples except P30 and P60 samples from 2 of the participants.

Complete blood counts were determined using whole blood samples at each time point. Total leukocyte count and the number of neutrophils, lymphocytes, monocytes, eosinophils, and basophils were analyzed using Sysmex XN analyzer (Sysmex Corporation, Kobe, Japan).

### Flow cytometry

**Figure 1:**
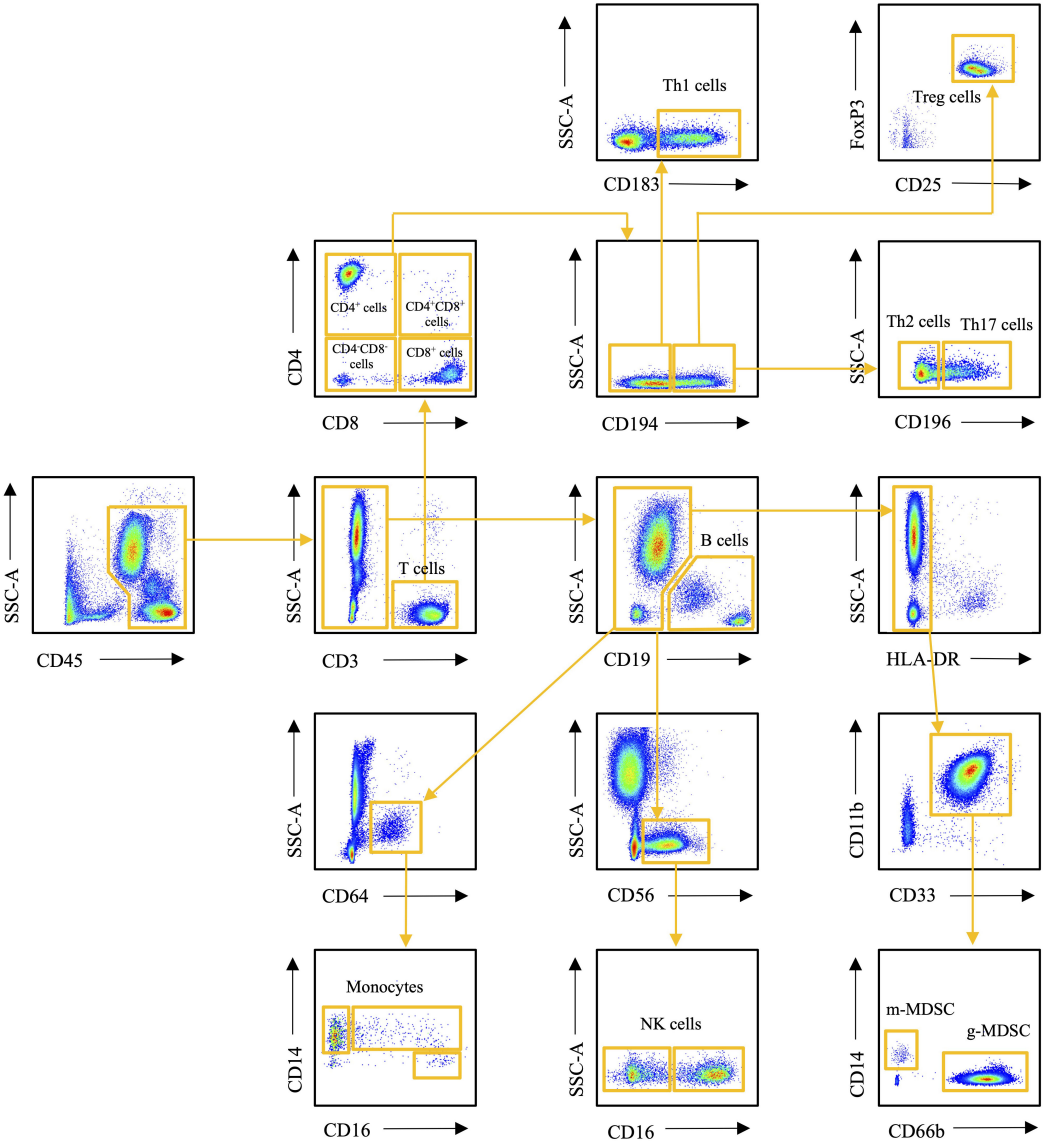
Flow cytometry gating strategy of the immune cells studied.

Flow cytometry was used to determine the number of circulating T cells, B cells, NK cells, monocyte subtypes and MDSCs. Fc Block (BD Biosciences, San Jose, USA) was added to 100 µL whole blood samples. The samples were stained with fluorophore-labeled monoclonal antibodies (BD Biosciences, San Jose, USA). The antibodies used were CD45-FITC (clone HI30), CD3-BV605 (clone UCHT1), CD4-BV421 (clone RPA-T4), CD8-BV786 (clone RPA-T8), CD196-APC (clone 11A9), CD183-PE (clone 1C6), CD194-PE-Cy7 (clone 1G1), and CD25-APC-Cy7 (clone M-A251) for staining panel 1, CD45-FITC (clone HI30), CD3-BV605 (clone UCHT1), CD19-PE-CY7 (clone HIB19), Anti-HLA-DR-APC-Cy7 (clone L243), CD33-BV711 (clone WM53), CD11b-BV421 (clone ICRF44), CD66b-PE (clone G10F5), and CD14-APC (clone M5E2) for staining panel 2, and CD45-FITC (clone HI30), CD14-APC (clone M5E2), CD16-PE (clone 3G8), CD19-PE-CY7 (clone HIB19), CD56-BV605 (clone NCAM16.2), and CD64-BV421 (clone 10.1) for staining panel 3. Red blood cells were lysed with 1X FACS solution (BD Biosciences, San Jose, USA) and samples were washed with phosphate buffered saline (PBS) (Thermo Fisher Scientific, Waltham, USA). Panel 1 samples were further processed for FoxP3 staining. For that, the samples were incubated with Fixation/Permeabilization working solution (Thermo Fisher Scientific, Waltham, USA) and washed with 1X Permeabilization buffer (Thermo Fisher Scientific, Waltham, USA). Samples were then incubated with FoxP3-antibody (clone 236A/E7) and washed with 1X Permeabilization buffer. 300 µL of PBS was added to each sample and samples were pipetted into a flat bottom 96-well plate. Cell counts were obtained by running 150 µL of each sample with a BD LSR Fortessa™ flow cytometer (BD Biosciences, San Jose, USA). All analyses were performed using FlowJo v10 software (FlowJo, LLC Ashland, Oregon, USA). The gating strategy is presented in **Figure 1**.

### Biomarker analysis

Characterization of breast cancer biomarkers was determined at Pathology Laboratory of Turku University Hospital as part of the patients’ routine diagnostics. Paraffin embedded cancer specimen were stained using Ventana Benchmark Ultra (Roche Diagnostics) for immunohistochemistry (IHC) with ER (SP1), PR (IE2), HER2 (4B5), and Ki-67 (30–9) antibodies (Ventana/Roche Diagnostics). Positive staining at HER2 IHC was always confirmed by *in situ* hybridization according to national and international guidelines (21). The degree of histological differentiation of carcinoma was determined according to Scaff-Bloom-Richardson (22, 23) and staging by the UICC TNM classification (24).

### Plasma volume change

The change in plasma volume (PV) during and after exercise bout was taken into consideration and calculated with following formula: ΔPV = (Hb_pre_*(1-Hct_post_))/(Hb_post_*(1-Hct_pre_))-1 (25). White blood cell (WBC) counts were adjusted to reflect the exercise-induced shifts in plasma volume with the following formula: WBC_corrected_ = WBC_uncorrected_*(1+ΔPV) (25).

### Statistical methods

After adjusting for plasma volume change, the number of immune cell populations determined by flow cytometry were corrected with the immune cell values obtained from the complete blood count. For the correction the following formula was used: WBC_corrected_ = total leukocyte count*WBC% of CD45^+^ cells. To determine the effect of acute exercise on immune cells counts and plasma volume, repeated measurement ANOVA was performed. When the main effect (time) was less than p <0.05, statistical differences in time points were considered by Tukey-*post-hoc* test. Paired samples t-test was used to compare means of heart rate, blood pressure, and lactate concentration at baseline and during exercise. The associations between immune cell mobilization and disease status and exercise intensity were examined by either Pearson´s or Spearman´s correlation. Sensitivity analyses were based on the visual inspection of the scatter blots. If we detected a possible outlier, its data was removed for the sensitivity analyses to determine whether the outlier affected the results. Significance was determined at p<0.05, and also at p<0.01 in correlation analyses. All statistical analyses were performed with Graphpad prism 8.0 and SAS 9.4.

## Results

### Participant characteristics

Characteristics of the study participants at baseline and their physiological response to exercise are presented in **Table 1**. Systolic and diastolic blood pressure, heart rate, and lactate concentration were significantly higher during exercise compared to baseline (p<0.0001 in all).

**Table 1:** Characteristics of the study participants (n=19).

Resting measurements	
Age, years	56 (8)
Height, cm	164.3 (4.5)
Weight, kg	76.9 (14.5)
BMI, kg/m^2^	28.9 (5.4)
Systolic blood pressure, mmHg	122 (16)
Diastolic blood pressure, mmHg	69 (7)
Heart rate, bpm	69 (10)
Lactate concentration, mmol/l	1.2 (0.5)
Disease status	
Grade I, %	16
Grade II, %	47
Grade III, %	37
ER+, %	95
PR+, %	89
HER2+, %	37
Tumor size, scale 1–3	2 (1)
Exercising measurements	
Heart rate at E15, bpm	128 (21)***
Heart rate at E30, bpm	138 (22)***
Heart rate % of maximal heart rate at E15, %	78 (13)
Heart rate % of maximal heart rate at E30, %	84 (14)
Systolic blood pressure at E15, mmHg	165 (33)***
Systolic blood pressure at E30, mmHg	165 (28)***
Diastolic blood pressure at E15, mmHg	109 (19)***
Diastolic blood pressure at E30, mmHg	114 (22)***
Lactate concentration at E15, mmol/l	3.6 (1.3)***
Lactate concentration at E30, mmol/l	3.8 (2.0)***
RPE at E15, Borg 6–20	12 (1)
RPE at E30, Borg 6–20	14 (2)
Ergometer´s workload, distance, km	15.9 (1.6)

Data presented as mean (SD).

Significant p-values; ***<0.001 as compared to rest.

BMI, body mass index; ER, estrogen receptor; PR, progesterone receptor; HER2, human epidermal growth factor receptor 2; RPE, rating of perceived exertion; E15, 15-minute timepoint during the exercise; E30, 30-minute timepoint during the exercise.

### Plasma volume

**Figure 2:**
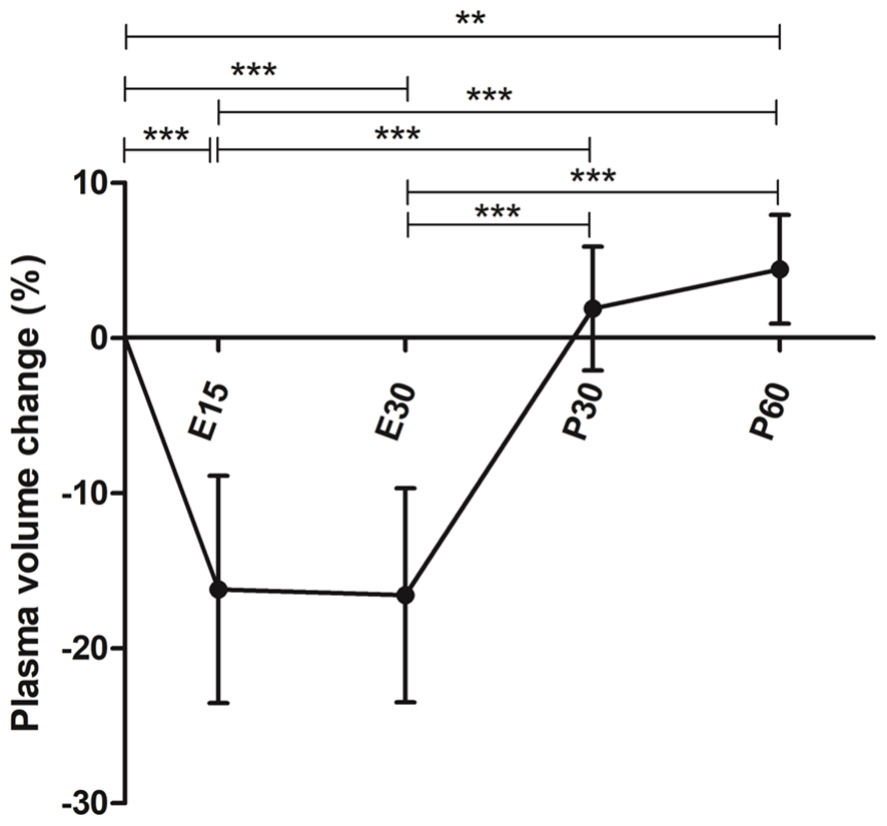
Mean plasma volume change during and after a 30-minute acute exercise. Error bars represent standard deviation. E15, 15-minute timepoint during the exercise; E30, 30-minute timepoint during the exercise; P30, 30 minutes post-exercise; P60, 60 minutes post-exercise. **p<0.01; ***p<0.001.

Mean reduction in plasma volume was 16.2% (SD 7.3) at 15-minute timepoint during exercise (E15) (p<0.0001) and 16.6% (SD 6.9) at 30-minute timepoint when they were finishing the exercise (E30) compared to baseline (p<0.0001). The plasma volume was 2.2% (SD 4.0) above baseline at 30-minutes post-exercise (P30) (p=0.2286) and 4.9% (SD 3.5) above baseline at 60-minutes post-exercise (P60) (p=0.0009) (**Figure 2**). Therefore, absolute number of white blood cells were adjusted for plasma volume change.

### Effect of acute exercise on different leukocyte subgroups

**Figure 3:**
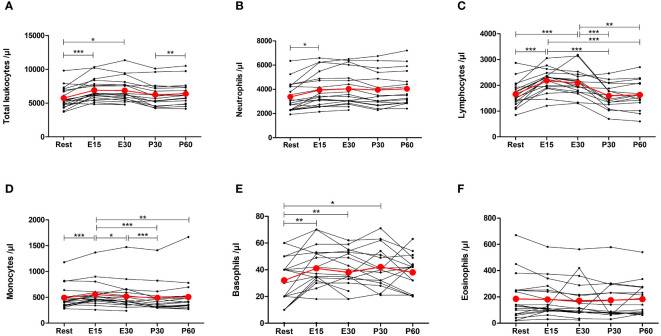
Exercise-induced changes in **(A)** total leukocytes, **(B)** neutrophils, **(C)** lymphocytes, **(D)** monocytes, **(E)** basophils, and **(F)** eosinophils. The number of total leukocytes, neutrophils, lymphocytes, monocytes, and basophils increased during exercise. The number of eosinophils did not change. Red lines represent the mean. E15, 15-minute timepoint during the exercise; E30, 30-minute timepoint during the exercise; P30, 30 minutes post-exercise; P60, 60 minutes post-exercise. *p<0.05; **p<0.01; ***p<0.001.

Acute exercise increased the number of total leukocytes by 22% (p<0.0001), neutrophils by 17% (p=0.0380), lymphocytes by 36% (p<0.0001), monocytes by 15% (p=0.0009), and basophils by 55% (p=0.0042) at E15 (**Figures 3A–E**). Increases in neutrophils and monocytes were observed only at E15 but the number of total leukocytes, lymphocytes, and basophils were also elevated at E30 compared to baseline (p=0.0234, p=0.0006, p=0.0070, respectively). Furthermore, the number of basophils was still elevated at P30 (p=0.0279). The number of eosinophils did not change (**Figure 3F**).

**Figure 4:**
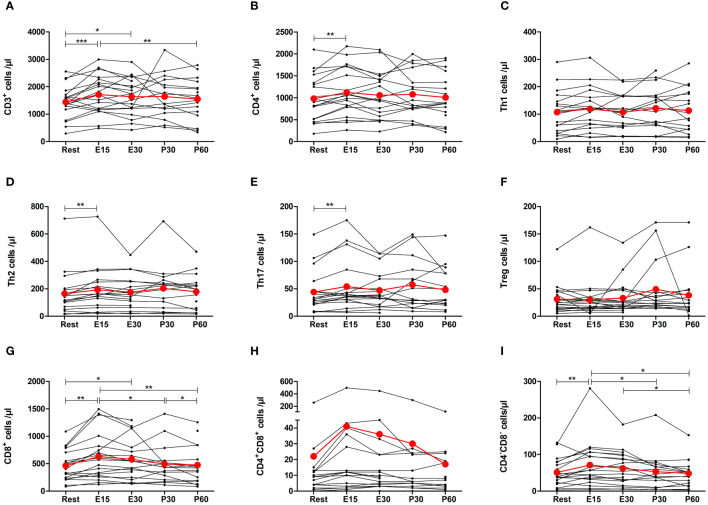
Exercise-induced changes in **(A)** total T cells, **(B)** CD4^+^ helper T cells, **(C)** Th1 cells, **(D)** Th2 cells, **(E)** Th17 cells, **(F)** regulatory T cells, **(G)** CD8^+^ cytotoxic T cells, **(H)** CD4^+^CD8^+^ T cells, and **(I)** CD4^-^CD8^-^ T cells. The number of total T cells, CD4^+^ T cells, Th2 cells, Th17 cells, CD8^+^ T cells, and CD4^-^CD8^-^ T cells increased during exercise. The number of Th1 cells, regulatory T cells, and CD4^+^CD8^+^ T cells did not change. Red lines represent the mean. E15, 15-minute timepoint during the exercise; E30, 30-minute timepoint during the exercise; P30, 30 minutes post-exercise; P60, 60 minutes post-exercise. *p<0.05; **p<0.01; ***p<0.001.

Total number of T cells increased by 27% at E15 compared to baseline (p=0.0002) and remained elevated at E30 (p=0.0242) before returning to baseline after the exercise (**Figure 4A**). The number of total CD4^+^ T helper (Th) cells increased only at E15 by 19% (p=0.0025) (**Figure 4B**). Further analysis of CD4^+^ cells showed that the number of Th1 cells increased 27% at E15 but it was not significant (**Figure 4C**). Th2 and Th17 cells increased by 24% (p=0.0013) and by 23% (p=0.0060) at E15, respectively (**Figures 4D, E**). There was no change in regulatory T cells (**Figure 4F**). The number of CD8^+^ cytotoxic T cells increased by 36% at E15 (p=0.0014), remained elevated at E30 (p=0.0139), and decreased to baseline after the exercise (**Figure 4G**). CD4^+^CD8^+^ double positive T cells did not change significantly, but the number of CD4^-^CD8^-^ double negative T cells increased by 43% at E15 (p=0.0397) and then returned to baseline (**Figures 4H, I**).

**Figure 5:**
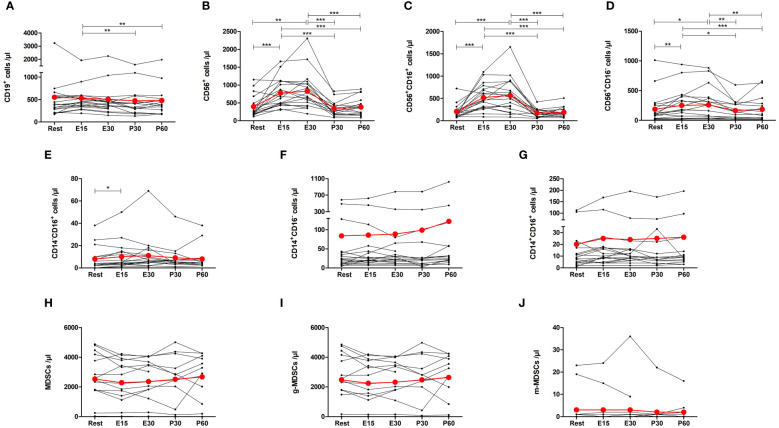
Exercise-induced changes in **(A)** CD19^+^ B cells, **(B)** total NK cells, **(C)** CD16^+^ NK cells, **(D)** CD16^-^ NK cells, **(E)** CD14^-^CD16^+^ monocytes, **(F)** CD14^+^CD16^-^ monocytes, **(G)** CD14^+^CD16^+^ monocytes, **(H)** total myeloid derived suppressor cells (MDSCs), **(I)** granulocytic MDSCs, and **(J)** monocytic MDSCs. The number of total NK cells, CD16^+^ NK cells, CD16^-^ NK cells, and CD14^-^CD16^+^ monocytes increased during exercise. The number of B cells decreased after exercise when compared to E15. The number of CD14^+^CD16^-^ monocytes, CD14^+^CD16^+^ monocytes, or MDSCs did not change. Red lines represent the mean. E15, 15-minute timepoint during the exercise; E30, 30-minute timepoint during the exercise; P30, 30 minutes post-exercise; P60, 60 minutes post-exercise. *p<0.05; **p<0.01; ***p<0.001.

The number of CD19^+^ B cells decreased by 18% at P30 (p=0.0084) and 17% at P60 (p=0.0084) compared to E15, but there was no significant change from baseline (**Figure 5A**). The number of total NK cells, CD16^+^ NK cells, and CD16^-^ NK cells increased by 154% (p=0.0001), 202% (p=0.0001) and 60% (p=0.0096) at E15, respectively, and remained elevated at E30 before returning to baseline (**Figures 5B–D**). Further, there was a 72% increase in the number of CD14^-^CD16^+^ monocytes at E15 compared to baseline (p=0.0178), but the number of CD14^+^CD16^-^ monocytes or CD14^+^CD16^+^ monocytes did not change (**Figures 5E–G**). The number of total MDSCs, g-MDSCs, or m-MDSCs did not change (**Figures 5H–J**).

In addition to absolute cell counts, immune cell proportions of total leukocytes were analyzed (**Table 2**). The proportion of neutrophils decreased at E15 (p=0.0227) and then increased at P30 and P60 compared to baseline (p=0.0015, p=0.0027, respectively). On the contrary, the proportion of lymphocytes increased at E15 (p=0.0003) and decreased at P30 and P60 compared to baseline (p=0.0242, p=0.0440, respectively). Monocyte proportion was below baseline at all time-points (p<0.05 in all) and eosinophil proportion was decreased only at E15 compared to baseline (p=0.0200). The proportion of Th2 cells increased at P30 compared to baseline (p=0.0375). The proportion of CD19^+^ B cells decreased at E15 compared to baseline (p=0.0397). The proportions of total NK cells and CD16^+^ NK cells increased at E15 (p=0.0006, p=0.0001, respectively) and at E30 (p=0.0031, p=0.0005, respectively) and decreased at P30 compared to baseline (p=0.0373, p=0.0347, respectively). Further, the proportions of total MDSCs and g-MDSCs decreased at E15 (p=0.0003, p=0.0003, respectively) and at E30 (p=0.0014, p=0.0015, respectively) compared to baseline. The proportions of other immune cells of total leukocytes remained unchanged in response to exercise (**Table 2**).

**Table 2:** Exercise-induced changes in immune cell proportions.

	Rest	E15	E30	P30	P60
% of total leukocytes					
Neutrophils	58.0 (7.6)	55.8 (8.5)*	58.1 (7.9)	61.6 (8.6)**	62.1 (8.6)**
Lymphocytes	29.7 (7.7)	32.8 (8.3)***	31.6 (7.7)	27.7 (8.1)*	27.2 (8.3)*
Monocytes	8.5 (2.7)	8.0 (2.4)*	7.4 (2.2)**	7.5 (2.3)***	7.6 (2.7)***
Eosinophils	3.2 (2.9)	2.7 (2.2)*	2.6 (2.3)	2.8 (2.5)	3.0 (2.4)
Basophils	0.6 (0.3)	0.6 (0.3)	0.6 (0.3)	0.7 (0.3)	0.6 (0.2)
CD3^+^	27.0 (11.4)	27.6 (12.0)	26.0 (11.0)	29.7 (12.5)	27.2 (12.4)
CD4^+^	17.1 (8.0)	16.7 (8.4)	15.9 (8.2)	18.3 (8.7)	16.9 (8.7)
Th1	1.8 (1.3)	1.7 (1.1)	1.6 (1.0)	1.8 (1.3)	1.7 (1.3)
Th2	2.7 (1.8)	2.7 (1.8)	2.6 (1.5)	3.3 (2.2)*	3.0 (1.9)
Th17	0.8 (0.5)	0.8 (0.6)	0.7 (0.5)	0.9 (0.7)	0.8 (0.6)
Treg	0.6 (0.5)	0.5 (0.5)	0.5 (0.5)	0.8 (0.9)	0.6 (0.7)
CD8^+^	8.7 (6.5)	9.5 (7.4)	8.8 (6.4)	10.1 (6.0)	8.8 (6.3)
CD4^+^CD8^+^	0.5 (1.6)	0.6 (1.9)	0.6 (1.8)	0.6 (1.8)	0.3 (0.7)
CD4^-^CD8^-^	0.9 (0.6)	1.0 (0.7)	0.9 (0.6)	1.0 (0.6)	0.8 (0.5)
CD19^+^	8.8 (6.6)	7.7 (3.8)*	7.4 (5.1)	7.3 (4.7)	7.3 (4.7)
CD56^+^	6.9 (5.1)	11.2 (4.7)***	11.7 (5.3)**	5.0 (2.5)*	5.8 (3.7)
CD56^+^CD16^+^	3.5 (1.8)	7.5 (3.7)***	7.9 (4.4)***	2.6 (1.0)*	2.9 (1.4)
CD56^+^CD16^-^	3.4 (4.7)	3.6 (3.8)	3.7 (3.7)	2.4 (2.2)	2.8 (3.2)
CD14^+^CD16^-^	1.3 (2.3)	1.1 (1.8)	1.0 (1.8)	1.1 (2.2)	1.4 (2.6)
CD14^+^CD16^+^	0.3 (0.4)	0.3 (0.4)	0.3 (0.4)	0.3 (0.5)	0.3 (0.5)
CD14^-^CD16^+^	0.1 (0.2)	0.1 (0.1)	0.1 (0.2)	0.1 (0.1)	0.1 (0.1)
MDSC	44.6 (26.5)	32.9 (20.8)***	34.8 (20.7)**	40.8 (27.9)	41.1 (25.6)
gMDSC	43.8 (26.7)	32.3 (20.9)***	34.3 (20.9)**	40.3 (27.9)	40.6 (25.6)
mMDSC	0.1 (0.1)	0.0 (0.1)	0.1 (0.2)	0.0 (0.1)	0.0 (0.1)

Data presented as mean (SD).

p-values; *<0.05, **<0.01, ***<0.001 as compared to rest.

E15, 15-minute timepoint during the exercise; E30, 30-minute timepoint during the exercise; P30, 30 minutes post-exercise; P60, 60 minutes post-exercise.

The proportions of different T cell subsets of total T cells and different helper T cells of total helper T cells were also examined (**Table 3**), and we found that the proportion of CD4^+^ T cells of total T cells decreased at E15 and E30 compared to baseline (p=0.0070, p=0.0181, respectively) whereas the proportion of CD8^+^ T cells of total T cells increased at E15 and E30 compared to baseline (p=0.0089, p=0.0348, respectively). The proportions of different helper T cells did not change (**Table 3**).

**Table 3:** Exercise-induced changes in T cell proportions.

	Rest	E15	E30	P30	P60
% of total T cells (CD3^+^)					
CD4^+^	64.5 (14.5)	61.6 (16.5)**	61.5 (16.2)*	63.7 (11.1)	65.3 (13.4)
CD8^+^	30.9 (14.5)	33.2 (15.8)**	33.2 (15.7)*	31.8 (11.4)	30.1 (13.2)
CD4^+^CD8^+^	2.6 (8.2)	3.4 (10.6)	3.24(10.6)	2.8 (9.6)	2.6 (8.3)
CD4^-^CD8^-^	3.3 (1.7)	3.7 (2.5)	3.7 (2.4)	3.2 (1.9)	3.1 (1.7)
% of helper T cells (CD4^+^)					
Th1	11.8 (8.1)	11.5 (7.5)	11.4 (7.5)	11.2 (7.6)	12.0 (8.5)
Th2	17.7 (14.5)	17.9 (14.4)	17.9 (13.1)	18.7 (16.4)	18.3 (15.5)
Th17	4.6 (2.9)	4.9 (3.5)	5.0 (3.7)	5.0 (3.5)	5.2 (4.4)
Treg	3.4 (2.3)	2.8 (2.1)	3.5 (2.8)	4.5 (4.4)	3.8 (3.5)

Data presented as mean (SD).

p-values; *<0.05, **<0.01 as compared to rest.

E15, 15-minute timepoint during the exercise; E30, 30-minute timepoint during the exercise; P30, 30 minutes post-exercise; P60, 60 minutes post-exercise.

CD8^+^/Treg and CD56^+^ NK cell/Treg ratios were also analyzed. CD8^+^/Treg ratio increased from 27 at baseline to 48 at E15 and NK cell/Treg ratio from 21 at baseline to 49 at E15 but the changes were not significant.

We also conducted correlation analyses between immune cell mobilization and exercise intensity and disease state that are found in **Supplementary Material**. Several associations were detected between cell mobilizations and disease state. For instance, tumor size correlated negatively with NK cell mobilization at E15, and progesterone receptor positivity correlated negatively with CD8^+^ T-cell mobilization.

## Discussion

In this study, the effect of 30-minute acute exercise on circulating immune cell populations was examined in newly diagnosed breast cancer patients. The absolute number of total leukocytes, neutrophils, lymphocytes, monocytes, basophils, total T cells, CD4^+^ T cells, Th2 cells, Th17 cells, CD8^+^ T cells, CD4^-^CD8^-^ T cells, NK cells, and CD14^-^CD16^+^ monocytes increased by exercise. The number of eosinophils, regulatory T cells, CD4^+^CD8^+^ T cells, B cells, MDSCs, CD14^+^CD16^+^, or CD14^+^CD16^-^ monocytes did not change during or after exercise compared to baseline. Furthermore, the proportions of total lymphocytes and NK cells of total leukocytes increased during exercise, while the proportions of neutrophils, monocytes, eosinophils, B cells, and MDSCs decreased. Overall, the profile of immune cells changed towards more cytotoxic/anti-tumorigenic by acute exercise.

The mobilization of immune cells seems to occur rapidly after the start of the exercise. However, the extent to which different immune cell subtypes are mobilized varies considerably (26). Here, both the absolute cell count and proportion of lymphocytes increased during exercise. In healthy individuals, NK cells are most sensitive to exercise stimuli due to their high number of adrenergic receptors (26–28). In this study, the increase in NK cells was also markedly greater than the increase of any other cell type. CD16^+^ NK cells increased by 202% at E30 compared to resting state, whereas the maximal increase in total lymphocytes was only 36%. The proportion of total NK cells and CD16^+^ NK cells of total leukocytes also increased during exercise. The increase in CD16^-^ NK cell count was lower than in CD16^+^ NK cells, only 60%, and the proportion of CD16^-^ NK cell did not change, which is in line with our previous studies with lymphoma and breast cancer patients (19, 29). Unexpectedly, no correlation between change in heart rate and NK cells was found in this study, although NK cell mobilization is known to be depend on exercise intensity via adrenergic signaling (26). There was, however, positive correlation between NK cell mobilization and change in lactate concentration. NK cell mobilization has also been studied in breast cancer survivors, where a significant increase was observed after acute exercise of 10x 3 minute intervals at 60% of VO2max (30). Further, in a recently published study, Schenk et al. (31) found that NK cell counts increase after acute exercise in prostate cancer patients. The settings of that study were similar to the current study; it was conducted before the start of cancer treatments and the intensity of the exercise (30-min at 75% of VO2max) was comparable as the heart rate percentage of maximal heart rate was 78 at E15 and 84 at E30 in the current study (32). The prognostic value of NK cells has been studied in many types of cancer including breast cancer (33). To date, the prognostic value of tumor-infiltrating NK cells is more evident, however, there are handful of studies also reporting that peripheral NK cell count independently predicts better survival in cancer patients (34–36).

Based on previous studies conducted in healthy individuals (26, 37), it was hypothesized that the increase in CD8^+^ T cells would be more pronounced than the increase in CD4^+^ T cells subsets. However, the 36% increase in CD8^+^ T cell count during exercise was only slightly greater than those in CD4^+^ T cells (19%), Th1 cells (27%), Th2 cells (24%), and Th17 cells (23%). The proportion of CD8^+^ T cells of total T cells, however, increased and the proportion of CD4^+^ T cells decreased during the exercise. Double negative CD4^-^CD8^-^ T cells also increased significantly by 43% during exercise. Some CD4^-^CD8^-^ T cells are γδ T cells which based on studies conducted with healthy individuals, have high response potential to acute exercise (26). Furthermore, absolute CD19^+^ B cell count did not change, and it has been previously described that B cells respond to adrenergic signaling to a lesser extent than NK cells and T cells (26), which is likely to explain these results. Based on previous studies in healthy individuals, it appears that lymphocytes with cytotoxic potential, hence NK cells, CD8^+^ T cells, and γδ T cells, are mobilized more than other lymphocytes during acute stress (38). In this study, the same pattern was observed in breast cancer patients. Comprehensive clinical significance of the mobilization of cytotoxic immune cells is yet to be investigated, but there is great evidence from preclinical studies that immune cell mobilization enhances tumor killing. Studies in animal models have found that epinephrine and IL-6 mediated mobilization of NK cells during exercise decreases tumor size (5), and that administration of lactate reduces tumor growth in cytotoxic T cell-dependent manner (6). It is not yet known whether exercise produces similar responses in cancer patients, but this study shows that cytotoxic immune cells are increased by exercise and that the mobilization of lymphocytes correlates with lactate concentration.

To the best of our knowledge, the effect of acute exercise on MDSCs has not been studied in humans before, including cancer patients. MDSCs are immunosuppressive cells that proliferate under chronic diseases such as cancer. In fact, cancer cells attract MDSCs to the tumor site where they enhance a pro-tumor microenvironment (39). In the present study, we found no change in the absolute number of total MDSCs, granulocytic MDSCs, or monocytic MDSCs after exercise. However, the proportions of total MDSCs and g-MDSCs decreased during exercise compared to baseline. Because MDSCs are pro-tumorigenic, it can be considered that a decrease in their proportion and on the other hand increase in cytotoxic T and NK cells changes the blood profile toward more cytotoxic and is thus beneficial for cancer patients. It is not known, however, if these changes in circulating blood correlate with changes in tumor microenvironment during and after exercise. Another pro-tumorigenic cell type is regulatory T cells, which inhibits the activity of effector T cells (40). Here we did not see any change in their number or proportion during or after exercise, which can again be a positive sign for cancer surveillance. Previously, regulatory T cell counts have been studied in patients with chronic lymphocytic leukemia (41). Those authors saw no change immediately after 45–60 minutes of cycling/running exercise, but a decrease was found 1 hour afterwards. In breast cancer survivors, the proportion of regulatory T cells is decreased 24 h after half marathon (42). A study conducted in mice finds that CD8^+^ T cell/regulatory T cell ratio doubled in breast cancer tumors after 8 weeks of training due to decreased number of regulatory T cells recruited to the tumor site (8). Further, another study with breast cancer mouse model finds that accumulation of MDSCs in tumor is reduced after 30 days of daily exercise when compared to sedentary control group (7). Such results are promising, but further investigation is needed to see whether these changes occur also in humans.

Granulocytes and monocytes have high functional diversity in tumor sites; some subtypes have pro-tumoral and some anti-tumoral effects (43–45). Therefore, it is difficult to predict whether their mobilization during exercise is beneficial to cancer patients. In the present study, the increase in absolute neutrophil count was significant only at E15 compared to baseline, which was unexpected since prolonged increase in neutrophils after exercise is commonly observed in healthy individuals (46, 47). The basophil count increased at E15 and was still elevated 30 minutes post-exercise. However, the proportion of basophils did not change. Oppositely, the absolute count of eosinophils did not change, but the proportion of eosinophils decreased at E15 compared to baseline. Exercise-induced changes in basophils and eosinophils have been studied relatively little in healthy individuals with inconsistent results (27, 47–49), and to the best of our knowledge, this is the first study to examine basophil and eosinophil responses to acute exercise in cancer patients. In breast cancer survivors, neutrophil, basophil, and eosinophil count clustered as granulocytes has been studied in response to half marathon, after which the granulocyte count increases but then decreases to baseline 24 h after exertion (42). However, it is well recognized that the intensity and duration of exercise affects the magnitude of immune cell mobilization (50–52), and thus a half marathon is very different stimulus compared to the 30-minute cycle ergometer exercise used in this study. Moreover, we found that total monocytes and non-classical (CD14^-^CD16^+^) monocytes increased at E15, but there was no change in classical or intermediate monocytes. The mobilization of non-classical monocytes, but not of classical or intermediate monocytes, have been linked to adrenergic activation and it has been suggested that they mobilize more compared to other monocyte subpopulations also in healthy individuals (26, 38). However, the number of intermediate (CD14^+^CD16^+^) and classical (CD14^+^CD16^-^) monocytes have been observed to increase after a 45-minute exercise bout in breast cancer survivors (53), and in our previous study, the number of intermediate monocytes, but not classical or non-classical monocytes was increased in breast cancer patients immediately after 10 minutes of exercise (29). Thus, the data are conflicting.

Based on the results from animal cancer models (5, 6), the immune cell mobilization due to exercise is believed to act as a key regulator in the positive effects of exercise on cancer. Thus, it is an encouraging non-pharmacologic strategy in cancer prevention and treatment with little to no adverse effects (1). In preclinical studies, exercise has also been shown to increase the effect of combined radiotherapy and immunotherapy in reducing tumor volume when compared to sedentary controls (7). Previous studies in healthy individuals combined with our results in breast cancer patients show that the mobilization of immune cells occurs fast and transiently after starting the exercise (54). In this study, the maximal increases in immune cell counts were all observed at E15, except for NK cells, which peaked at E30. For many subpopulations, the number decreased back to baseline at the end of the exercise. In fact, it is suggested that the positive effects of immune cell modulation by exercise occurs during and soon after exercise and with regular exercise, the effect accumulates (55). Although the phenomenon is well studied in healthy individuals, exercise-induced mobilization may not be exactly similar in people having chronic diseases, such as cancer. Breast cancer type and disease stage may contribute to the magnitude of this phenomenon and therefore we also analyzed associations between well-established biomarkers and immune cell mobilization. We found that CD8^+^ T cell mobilization at E15 correlated negatively with ER positivity and that total T cell and CD8^+^ T cell mobilization correlated negatively with PR positivity both at E15 and at E30. Interestingly, similar significant correlation between ER positivity and CD8^+^ T cells were found in our recent study where breast cancer patients did a 10-minute acute cycling exercise with light-to-moderate exercise intensity (29). The explanation for these observations is however unknown, but it may appear that cancer status indeed affects these responses. However, we also acknowledge that some correlations can be just random findings. In fact, when significance level of the p-value was set to 0.01, only the correlation between PR positivity and CD8^+^ T cell mobilization remained significant. No difference have been observed in the number of circulating T cells between different breast cancer subtypes, but HER2-positive and triple negative breast cancers typically have higher level of lymphocytes infiltrated in the tumor than hormone receptor positive cancers (56). This area warrants further studies to explore whether cancer status affects exercise-induced immune cell mobilization.

The study has certain strengths and limitations. The biggest strength of our study was the timing of the consecutive blood sampling, especially the two samples during the exercise. If blood samples would have been drawn only pre- and immediately post-exercise, a lot of information would have been lost as many of the changes were transient. Indeed, the majority of the significant changes were observed half-way during the exercise. Second, we analyzed complete blood counts and calculated plasma volume change during the exercise. Therefore, we were able to correct the effect of plasma volume change on immune cell concentrations. However, it is known that intensity of exercise has an effect on immune cell mobilization, but we did not control the intensity in a standardized manner that could enable the same relative exercise intensity for each patient. This could have led to variance in responses to exercise. Controlling this would have required maximal fitness testing, which would have been time-consuming and stressful. This study was conducted in a short window between cancer diagnosis and treatments making it also challenging to schedule the maximal fitness tests and perform the study protocol. However, all patients reached the intended 70% of maximal heart rate goal, and the patients classified the rate of exertion during the exercise bout as moderate on a Borg scale with very small standard deviations. As a strength, the exercise itself represented a real-world situation as the subjects were allowed to exercise as they would exercise in their daily living. Further, a correlation analysis was performed to examine the influence of exercise intensity as well as disease status on immune cell mobilization in these participants. Given that our correlation values were not particularly strong, and p-values were mostly just below 0.05, we acknowledge that we must be precautionary with the interpretations of these associations. These analyses are explorative in nature and findings must be repeated in other studies.

## Conclusions

The present study aimed to examine changes in circulating immune cell counts in breast cancer patients conducting acute cycling exercise before onset of a curative oncologic treatment. We found that 30-min acute exercise is sufficient to mobilize total leukocytes, neutrophils, lymphocytes, monocytes, basophils, total T cells, CD4^+^ T cells, Th 2 cells, Th 17 cells, CD8^+^ T cells, CD4^-^CD8^-^ T cells, NK cells, and CD14^-^CD16^+^ monocytes. Additionally, our findings suggest that the profile of immune cells change towards more cytotoxic/anti-tumorigenic as the proportions of CD8^+^ T cells and NK cells increased and the proportion of MDSCs proportion decreased in breast cancer patients with 30-minute exercise. Further studies are however needed to examine the clinical significance of this exercise-induced immune cell mobilization and they also should focus on the function of pro-tumor and anti-tumor immune cells in the context of exercise and cancer and whether cancer status affects exercise-induced immune cell mobilization.

## Data availability statement

The raw data supporting the conclusions of this article will be made available by the authors, without undue reservation.

## Ethics statement

The studies involving humans were approved by Ethics Committee of the Hospital District of Southwestern Finland. The studies were conducted in accordance with the local legislation and institutional requirements. The participants provided their written informed consent to participate in this study.

## Author contributions

TK: Data curation, Formal analysis, Investigation, Writing – original draft. SL: Investigation, Writing – review & editing. JNe: Investigation, Writing – review & editing. JNo: Investigation, Writing – review & editing. MH: Conceptualization, Writing – review & editing. CS: Writing – review & editing. HR: Conceptualization, Methodology, Writing – review & editing. HM: Conceptualization, Writing – review & editing. PR: Conceptualization, Methodology, Writing – review & editing. IH: Conceptualization, Funding acquisition, Writing – review & editing.
