# Supplementary material for: The effect of exercise and disease status on mobilization of anti-tumorigenic and pro-tumorigenic immune cells in women with breast cancer
Source: Front Immunol. 2024 Jun 24;15:1394420. doi: 10.3389/fimmu.2024.1394420 (PMC11228136; doi:10.3389/fimmu.2024.1394420)
Supplement: Supplementary file 1 [file Table_1.docx]

**Supplementary material**

**The effect of exercise and disease status on mobilization of anti-tumorigenic and pro-tumorigenic immune cells in women with breast cancer**

Tiia Koivula, Salla Lempiäinen, Joona Neuvonen, Jooa Norha, Maija Hollmén, Carl Johan Sundberg, Helene Rundqvist, Heikki Minn, Petteri Rinne, Ilkka Heinonen

**Results**

***Correlations between exercise intensity and immune cell mobilization***

Correlations between exercise intensity and the immune cell mobilization are presented in Tables 1 and 2 for E15 and E30, respectively. Heart rate (HR), heart rate percentage of age-predicted maximal heart rate (HR% of HRmax), and energy expenditure (EE) at E15 correlated positively with the change in basophils, total monocytes, and CD14^-^CD16^+^ monocytes. Further, mean arterial pressure (MAP) correlated positively with the change in CD16^-^ NK cells. (Table 1). At E30, HR and HR% of HRmax correlated positively with the changes in monocytes. Rate pressure product at E30 correlated positively with the lymphocyte and basophil mobilization. MAP at E30 correlated positively with the change in basophils. Additionally, lactate concentration at E30 correlated positively with the change in lymphocytes and CD16^+^ NK cells. (Table 2) We also did sensitivity analyses for the data, after which the correlation between lactate concentration and the change in CD16^+^ NK cells strengthened to r=0,8020 (p<0,0001). In addition correlation between lactate concentration at E30 and the change in total NK cells turned significant (r=0.7668, p=0.0002). When statistical significance level was set to 0.01 to take into account the large number of tests, correlations between HR and HR% of HRmax at E15 and the change in total and CD14^-^CD16^+^ monocytes, and between EE at E15 and CD14^-^CD16^+^ monocytes, and between MAP at E30 and basophil mobilization, and between lactate concentration at E30 and total and CD16^+^ NK cell mobilization remained significant.

***Correlations between disease state and immune cell mobilization***

Correlations between disease status and the mobilization of immune cells at E15 and E30 are presented in Tables 3 and 4, respectively. Cancer grade correlated positively with the change in basophils at E15. Tumor size correlated negatively with the change in CD4^-^CD8^-^ T cells, total NK cells and CD16^+^ NK cells at E15. Lymph node involvement correlated negatively with the change in Th17 cells at E15. Estrogen receptor (ER) positivity correlated negatively with the change in CD8^+^ T cells at E15. Progesterone receptor (PR) positivity correlated negatively with the change in basophils, total T cells, and CD8^+^ T cells at E15, and with CD8^+^ T cells at E30. HER2 positivity correlated negatively with the change in total NK cells and CD16^+^ NK cells at E15. After sensitivity analyses the correlation between tumor size and the change in CD4^-^CD8^-^ T cells turned insignificant. Further, when statistical significance level was set to 0.01, only the correlations between cancer grade and the change in basophils at E15 and PR positivity and change in CD8^+^ T cells at E30 remained significant.

| **Table 1. Correlations between exercise intensity and immune cell mobilization at E15.** | | | | | |  |  |  |  |  |  |
| --- | --- | --- | --- | --- | --- | --- | --- | --- | --- | --- | --- |
|  | HR,  bpm | HR% of  HR max | RPP,  bpm*mmHg | MAP,  mmHg | Lactate,  mmol/l | | | EE,  kcal*min^-1^ | | | |
| Total leukocytes | 0.29 | 0.37 | 0.13 | -0.30 | 0.17 | | | 0.24 | | | |
| Neutrophils | 0.09 | 0.17 | -0.08 | -0.46 | 0.07 | | | 0.05 | | | |
| Lymphocytes | 0.36 | 0.43 | 0.32 | 0.05 | 0.23 | | | 0.31 | | | |
| Monocytes | **0.60**** | **0.65**** | 0.48 | -0.11 | 0.35 | | | **0.51*** | | | |
| Eosinophils | **0.57*** | **0.56*** | 0.27 | -0.12 | 0.18 | | | **0.51*** | | | |
| Basophils | **0.50*** | **0.54*** | 0.28 | -0.05 | 0.39 | | | **0.53*** | | | |
| CD3^+^ | -0.01 | 0.01 | 0.14 | 0.18 | -0.29 | | | 0.03 | | | |
| CD4^+^ | -0.08 | -0.05 | 0.18 | 0.29 | -0.18 | | | -0.04 | | | |
| Th1 | 0.001 | 0.06 | -0.02 | 0.37 | 0.08 | | | 0.03 | | | |
| Th2 | 0.02 | 0.08 | 0.03 | 0.27 | 0.03 | | | 0.06 | | | |
| Th17 | 0.11 | 0.16 | -0.12 | 0.15 | 0.03 | | | 0.13 | | | |
| Treg | 0.12 | 0.14 | 0.04 | 0.01 | 0.19 | | | 0.09 | | | |
| CD8^+^ | 0.08 | 0.09 | 0.05 | -0.05 | -0.30 | | | 0.12 | | | |
| CD4^+^CD8^+^ | 0.23 | 0.36 | -0.05 | -0.26 | **0.48*** | | | 0.10 | | | |
| CD4^-^CD8^-^ | -0.05 | -0.07 | 0.14 | 0.37 | 0.001 | | | -0.08 | | | |
| CD19^+^ | 0.20 | 0.19 | 0.03 | -0.23 | 0.11 | | | 0.23 | | | |
| CD56^+^ | 0.18 | 0.22 | 0.27 | 0.30 | 0.41 | | | 0.09 | | | |
| CD56^+^CD16^+^ | 0.17 | 0.22 | 0.19 | 0.16 | 0.44 | | | 0.08 | | | |
| CD56^+^CD16^-^ | 0.15 | 0.15 | 0.41 | **0.60*** | 0.21 | | | 0.09 | | | |
| CD14^+^CD16^-^ | **0.55*** | **0.51*** | 0.47 | 0.20 | 0.03 | | | **0.53*** | | | |
| CD14^+^CD16^+^ | 0.34 | 0.42 | 0.28 | -0.11 | 0.14 | | | 0.35 | | | |
| CD14^-^CD16^+^ | **0.61**** | **0.68**** | 0.43 | 0.05 | 0.36 | | | **0.60**** | | | |
| MDSC | 0.05 | 0.02 | 0.03 | 0.001 | -0.20 | | | 0.09 | | | |
| g-MDSC | 0.05 | 0.01 | 0.04 | 0.02 | -0.21 | | | 0.08 | | | |
| m-MDSC | -0.40 | -0.19 | -0.18 | 0.25 | -0.01 | | | -0.43 | | | |
| p-values; *<0.05, **<0.01. | | | | | | | | |  |  |  |
| Abbreviations; HR= heart rate, RPP= rate pressure product, MAP= mean arterial pressure, EE= energy expenditure. | | | | | | | | |  |  |  |

| **Table 2. Correlations between exercise intensity and immune cell mobilization at E30.** | | | | | | |  |  |  |  |  |  |  |
| --- | --- | --- | --- | --- | --- | --- | --- | --- | --- | --- | --- | --- | --- |
|  | HR,  bpm | HR% of  HR max | RPP,  bpm*mmHg | MAP,  mmHg | Lactate,  mmol/l | EE,  kcal*min^-1^ | | | | Workload,  distance | | | |
| Total leukocytes | 0.31 | 0.36 | 0.44 | 0.24 | 0.38 | 0.26 | | | | 0.33 | | | |
| Neutrophils | 0.15 | 0.18 | 0.04 | -0.03 | 0.22 | 0.08 | | | | 0.25 | | | |
| Lymphocytes | 0.34 | 0.43 | **0.58*** | 0.43 | **0.50*** | 0.37 | | | | 0.27 | | | |
| Monocytes | **0.51*** | **0.52*** | 0.47 | -0.12 | 0.19 | 0.40 | | | | 0.43 | | | |
| Eosinophils | 0.12 | 0.15 | 0.40 | 0.38 | 0.11 | 0.17 | | | | **0.52*** | | | |
| Basophils | 0.29 | 0.40 | **0.61*** | **0.68**** | 0.43 | 0.39 | | | | 0.13 | | | |
| CD3^+^ | 0.20 | 0.24 | 0.30 | 0.32 | 0.18 | 0.29 | | | | 0.13 | | | |
| CD4^+^ | 0.12 | 0.14 | 0.35 | 0.46 | 0.17 | 0.18 | | | | 0.21 | | | |
| Th1 | 0.19 | 0.22 | 0.21 | 0.19 | 0.13 | 0.15 | | | | -0.22 | | | |
| Th2 | 0.24 | 0.23 | 0.37 | 0.37 | 0.10 | 0.18 | | | | 0.20 | | | |
| Th17 | 0.39 | 0.39 | 0.52 | 0.36 | 0.22 | 0.26 | | | | -0.08 | | | |
| Treg | 0.001 | 0.04 | 0.08 | 0.20 | -0.17 | -0.05 | | | | -0.07 | | | |
| CD8^+^ | 0.25 | 0.30 | 0.15 | 0.16 | 0.14 | 0.35 | | | | -0.05 | | | |
| CD4^+^CD8^+^ | 0.37 | **0.51*** | 0.34 | 0.04 | **0.57*** | 0.11 | | | | -0.04 | | | |
| CD4^-^CD8^-^ | 0.16 | 0.12 | -0.001 | -0.23 | 0.23 | 0.09 | | | | 0.32 | | | |
| CD19^+^ | 0.25 | 0.23 | 0.33 | 0.24 | -0.03 | 0.16 | | | | 0.25 | | | |
| CD56^+^ | -0.11 | -0.06 | -0.14 | -0.07 | 0.41 | -0.08 | | | | 0.02 | | | |
| CD56^+^CD16^+^ | -0.07 | -0.01 | -0.08 | 0.001 | **0.50*** | -0.07 | | | | -0.03 | | | |
| CD56^+^CD16^-^ | -0.19 | -0.18 | -0.28 | -0.24 | 0.09 | -0.11 | | | | 0.17 | | | |
| CD14^+^CD16^-^ | 0.38 | 0.39 | 0.28 | -0.30 | -0.03 | 0.42 | | | | 0.08 | | | |
| CD14^+^CD16^+^ | 0.36 | 0.39 | 0.35 | -0.30 | 0.15 | 0.44 | | | | -0.001 | | | |
| CD14^-^CD16^+^ | 0.26 | 0.31 | 0.01 | -0.28 | 0.22 | 0.38 | | | | -0.09 | | | |
| MDSC | 0.22 | 0.16 | -0.40 | -0.51 | 0.10 | 0.15 | | | | -0.15 | | | |
| g-MDSC | 0.20 | 0.14 | -0.43 | -0.54 | 0.10 | 0.14 | | | | -0.16 | | | |
| m-MDSC | -0.30 | -0.12 | -0.34 | -0.10 | -0.01 | -0.40 | | | | -0.02 | | | |
| p-values; *<0.05, **<0.01. | | | | | | | | | |  |  |  |  |
| Abbreviations; HR= heart rate, RPP= rate pressure product, MAP= mean arterial pressure,  EE= energy expenditure. | | | | | | | | | |  |  |  |  |

| **Table 3. Correlations between disease status and immune cell mobilization at E15.** | | | | | |  |  |  |  |  |  |  |
| --- | --- | --- | --- | --- | --- | --- | --- | --- | --- | --- | --- | --- |
|  | Grade,  scale 1–3 | Tumor size,  scale 1–3 | LN  involvement,  scale 1–3 | ER,  % | PR,  % | HER2,  pos/neg | | | | Ki-67,  % | | |
| Total leukocytes | 0.28 | -0.14 | -0.001 | -022 | -0.37 | 0.09 | | | | 0.22 | | |
| Neutrophils | 0.28 | -0.15 | -0.29 | -0.24 | -0.30 | 0.22 | | | | 0.40 | | |
| Lymphocytes | 0.15 | -0.27 | -0.01 | -0.11 | -0.36 | -0.06 | | | | -0.16 | | |
| Monocytes | 0.20 | -0.29 | -0.04 | 0.001 | -0.15 | 0.31 | | | | 0.07 | | |
| Eosinophils | 0.28 | 0.11 | 0.29 | -0.09 | -0.13 | 0.25 | | | | 0.10 | | |
| Basophils | **0.59**** | -0.07 | 0.09 | -0.08 | **-0.47*** | 0.06 | | | | 0.22 | | |
| CD3^+^ | 0.17 | 0.04 | 0.19 | -0.39 | **-0.47*** | 0.19 | | | | -0.18 | | |
| CD4^+^ | 0.05 | -0.12 | 0.12 | -0.07 | -0.22 | -0.13 | | | | -0.24 | | |
| Th1 | -0.18 | -0.25 | -0.16 | 0.14 | -0.16 | -0.19 | | | | -0.07 | | |
| Th2 | -0.02 | -0.41 | -0.23 | 0.17 | -0.15 | -0.27 | | | | -0.22 | | |
| Th17 | -0.27 | -0.30 | **-0.48*** | 0.24 | -0.40 | -0.04 | | | | -0.27 | | |
| Treg | -0.02 | 0.01 | -0.18 | 0.37 | 0.12 | 0.001 | | | | -0.10 | | |
| CD8^+^ | 0.25 | -0.12 | 0.04 | **-0.53*** | **-0.56*** | 0.28 | | | | -0.06 | | |
| CD4^+^CD8^+^ | -0.12 | -0.46 | -0.29 | 0.03 | -0.31 | -0.03 | | | | -0.10 | | |
| CD4^-^CD8^-^ | 0.15 | **-0.47*** | -0.23 | -0.09 | -0.07 | -0.25 | | | | -0.07 | | |
| CD19^+^ | -0.04 | 0.08 | 0.07 | -0.12 | -0.09 | 0.09 | | | | 0.11 | | |
| CD56^+^ | -0.29 | **-0.53*** | -0.14 | 0.25 | 0.09 | **-0.53*** | | | | -0.14 | | |
| CD56^+^CD16^+^ | -0.29 | **-0.49*** | -0.08 | 0.23 | -0.01 | **-0.56*** | | | | -0.13 | | |
| CD56^+^CD16^-^ | -0.19 | -0.41 | -0.19 | 0.23 | 0.34 | -0.25 | | | | -0.12 | | |
| CD14^+^CD16^-^ | 0.15 | -0.16 | -0.29 | 0.001 | -0.22 | 0.34 | | | | 0.34 | | |
| CD14^+^CD16^+^ | -0.16 | 0.01 | 0.03 | 0.20 | -0.10 | 0.11 | | | | 0.22 | | |
| CD14^-^CD16^+^ | 0.04 | -0.07 | 0.15 | 0.35 | 0.02 | -0.17 | | | | 0.05 | | |
| MDSC | 0.31 | -0.03 | -0.16 | **-0.59*** | -0.37 | 0.52 | | | | 0.23 | | |
| g-MDSC | 0.23 | -0.09 | -0.13 | **-0.59*** | -0.37 | 0.52 | | | | 0.23 | | |
| m-MDSC | -0.25 | -0.31 | -0.23 | -0.01 | -0.12 | -0.31 | | | | **-0.52*** | | |
| p-values; *<0.05, **<0.01. | | | | | |  |  |  |  |  |  |  |
| Abbreviations; LN= lymph node, ER= estrogen receptor, PR= progesterone receptor, HER2= human epidermal growth factor receptor 2, Ki-67= proliferation index. | | | | | |  |  |  |  |  |  |  |

| **Table 4. Correlations between disease status and immune cell mobilization at E30.** | | | | | |  |  |  |  |  |  |  |
| --- | --- | --- | --- | --- | --- | --- | --- | --- | --- | --- | --- | --- |
|  | Grade,  scale 1–3 | Tumor size,  scale 1–3 | LN  involvement,  scale 1–3 | ER,  % | PR,  % | HER2,  pos/neg | | | | Ki-67,  % | | |
| Total leukocytes | 0.38 | -0.07 | 0.02 | -0.09 | -0.32 | 0.09 | | | | 0.36 | | |
| Neutrophils | **0.49*** | -0.02 | -0.08 | -0.25 | -0.30 | 0.19 | | | | **0.52*** | | |
| Lymphocytes | 0.02 | -0.20 | 0.12 | 0.16 | -0.29 | -0.19 | | | | -0.03 | | |
| Monocytes | 0.02 | -0.28 | -0.13 | 0.10 | -0.04 | 0.03 | | | | 0.42 | | |
| Eosinophils | 0.11 | 0.13 | 0.19 | 0.001 | 0.17 | 0.001 | | | | -0.12 | | |
| Basophils | 0.43 | 0.16 | 0.31 | 0.02 | -0.38 | 0.08 | | | | 0.09 | | |
| CD3^+^ | 0.37 | 0.30 | 0.39 | -0.24 | **-0.53*** | 0.41 | | | | 0.19 | | |
| CD4^+^ | 0.19 | 0.38 | 0.42 | -0.12 | -0.35 | 0.28 | | | | 0.12 | | |
| Th1 | -0.09 | 0.17 | -0.07 | 0.05 | -0.37 | 0.15 | | | | -0.17 | | |
| Th2 | 0.17 | 0.11 | 0.03 | -0.02 | -0.20 | 0.15 | | | | 0.11 | | |
| Th17 | 0.11 | 0.02 | -0.10 | 0.02 | **-0.50*** | 0.17 | | | | 0.04 | | |
| Treg | 0.28 | 0.26 | 0.09 | -0.45 | **-0.47*** | 0.44 | | | | 0.25 | | |
| CD8^+^ | 0.36 | 0.15 | 0.29 | -0.29 | **-0.59**** | 0.38 | | | | 0.21 | | |
| CD4^+^CD8^+^ | -0.30 | -0.23 | -0.09 | 0.07 | -0.28 | -0.03 | | | | -0.10 | | |
| CD4^-^CD8^-^ | 0.15 | -0.38 | -0.14 | -0.14 | -0.29 | -0.25 | | | | 0.05 | | |
| CD19^+^ | 0.05 | -0.16 | 0.001 | -0.06 | -0.03 | -0.28 | | | | 0.06 | | |
| CD56^+^ | -0.24 | -0.35 | -0.10 | 0.25 | 0.08 | -0.34 | | | | -0.01 | | |
| CD56^+^CD16^+^ | -0.18 | -0.30 | 0.02 | 0.21 | -0.02 | -0.31 | | | | -0.001 | | |
| CD56^+^CD16^-^ | -0.32 | -0.30 | -0.07 | 0.30 | 0.33 | -0.28 | | | | -0.04 | | |
| CD14^+^CD16^-^ | 0.09 | -0.13 | -0.21 | 0.04 | -0.33 | 0.22 | | | | 0.33 | | |
| CD14^+^CD16^+^ | 0.16 | -0.14 | 0.001 | 0.12 | -0.29 | 0.13 | | | | 0.33 | | |
| CD14^-^CD16^+^ | 0.04 | -0.20 | 0.10 | 0.23 | -0.12 | -0.13 | | | | 0.24 | | |
| MDSC | 0.49 | 0.02 | 0.01 | -0.39 | -0.32 | 0.26 | | | | 0.20 | | |
| g-MDSC | **0.54*** | -0.04 | 0.05 | -0.40 | -0.33 | 0.26 | | | | 0.20 | | |
| m-MDSC | **-0.54*** | 0.02 | -0.29 | 0.05 | 0.05 | -0.13 | | | | -0.39 | | |
| p-values; *<0.05, **<0.01. | | | | | |  |  |  |  |  |  |  |
| Abbreviations; LN= lymph node, ER= estrogen receptor, PR= progesterone receptor, HER2= human epidermal growth factor receptor 2, Ki-67= proliferation index. | | | | | |  |  |  |  |  |  |  |
